# Exploring protein lipidation by mass spectrometry-based proteomics

**DOI:** 10.1093/jb/mvad109

**Published:** 2023-12-15

**Authors:** Kazuya Tsumagari, Yosuke Isobe, Koshi Imami, Makoto Arita

**Affiliations:** Proteome Homeostasis Research Unit, RIKEN Center for Integrative Medical Sciences, 1-7-22 Suehiro-cho, Tsurumi-ku, Yokohama, Kanagawa 230-0045, Japan; Laboratory for Metabolomics, RIKEN Center for Integrative Medical Sciences, 1-7-22 Suehiro-cho, Tsurumi-ku, Yokohama, Kanagawa 230-0045, Japan; Laboratory for Integrative Genomics, RIKEN Center for Integrative Medical Sciences, 1-7-22 Suehiro-cho, Tsurumi-ku, Yokohama, Kanagawa 230-0045, Japan; Laboratory for Metabolomics, RIKEN Center for Integrative Medical Sciences, 1-7-22 Suehiro-cho, Tsurumi-ku, Yokohama, Kanagawa 230-0045, Japan; Division of Physiological Chemistry and Metabolism, Graduate School of Pharmaceutical Sciences, Keio University, 1-5-30 Shibakoen, Minato-ku, Tokyo 105-8512, Japan; Cellular and Molecular Epigenetics Laboratory, Graduate School of Medical Life Science, Yokohama City University, 1-7-29 Suehiro-cho, Tsurumi-ku, Yokohama, Kanagawa 230-0045, Japan; Proteome Homeostasis Research Unit, RIKEN Center for Integrative Medical Sciences, 1-7-22 Suehiro-cho, Tsurumi-ku, Yokohama, Kanagawa 230-0045, Japan; Laboratory for Metabolomics, RIKEN Center for Integrative Medical Sciences, 1-7-22 Suehiro-cho, Tsurumi-ku, Yokohama, Kanagawa 230-0045, Japan; Laboratory for Integrative Genomics, RIKEN Center for Integrative Medical Sciences, 1-7-22 Suehiro-cho, Tsurumi-ku, Yokohama, Kanagawa 230-0045, Japan; Laboratory for Metabolomics, RIKEN Center for Integrative Medical Sciences, 1-7-22 Suehiro-cho, Tsurumi-ku, Yokohama, Kanagawa 230-0045, Japan; Division of Physiological Chemistry and Metabolism, Graduate School of Pharmaceutical Sciences, Keio University, 1-5-30 Shibakoen, Minato-ku, Tokyo 105-8512, Japan; Cellular and Molecular Epigenetics Laboratory, Graduate School of Medical Life Science, Yokohama City University, 1-7-29 Suehiro-cho, Tsurumi-ku, Yokohama, Kanagawa 230-0045, Japan; Human Biology-Microbiome-Quantum Research Center (WPI-Bio2Q), Keio University, 35 Shinanomachi, Shinjuku-ku, Tokyo 160-8582, Japan

**Keywords:** chemical probe, lipidation, mass spectrometry, post-translational modification, proteomics

## Abstract

Protein lipidation is a common co- or post-translational modification that plays a crucial role in regulating the localization, interaction and function of cellular proteins. Dysregulation of lipid modifications can lead to various diseases, including cancer, neurodegenerative diseases and infectious diseases. Therefore, the identification of proteins undergoing lipidation and their lipidation sites should provide insights into many aspects of lipid biology, as well as providing potential targets for therapeutic strategies. Bottom-up proteomics using liquid chromatography/tandem mass spectrometry is a powerful technique for the global analysis of protein lipidation. Here, we review proteomic methods for profiling protein lipidation, focusing on the two major approaches: the use of chemical probes, such as lipid alkyne probes, and the use of enrichment techniques for endogenous lipid-modified peptides. The challenges facing these methods and the prospects for developing them further to achieve a comprehensive analysis of lipid modifications are discussed.

## Graphical Abstract

**Graphical Abstract:**
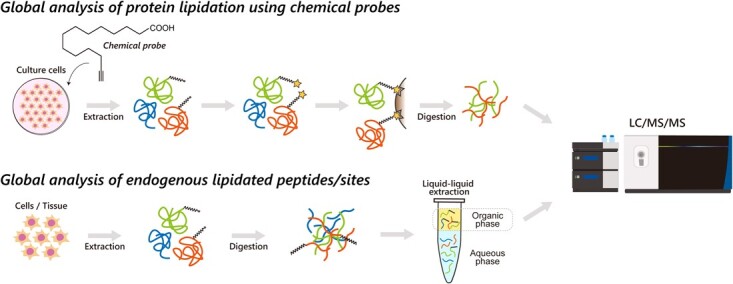

Proteins undergo covalent modification by a variety of lipids, including fatty acids, isoprenoids and phospholipids, in processes collectively referred to as lipidation *(**1**)*. Protein lipidation is common in living organisms, including plants, yeast and mammals *(**2**,**3**)*. The interplay between lipid types and the target sites/motifs for lipid modification is tightly regulated (Table 1). For instance, both the α-amino group of glycine at the N-terminus of proteins having the consensus motif G*XXX*S/T (where *X* can be any amino acid except proline) and the side chain of lysine residues of some proteins are myristoylated by *N*-myristoyl transferases (NMTs) *(**4**,**5**)*. The side chain of cysteine residues can undergo palmitoylation mediated by palmitoyl acyltransferases (ZDHHCs) *(**6**)*. Prenylations, such as farnesylation or geranylgeranylation, occur at the side chain of cysteine within a C*AAX* box motif (where C and *A* represent cysteine and an aliphatic amino acid, respectively) located at the protein C-terminus, followed by the proteolytic removal of the last three amino acids of the protein (*AAX*) and the capping of the C-terminus by methylation *(**7**)*. In addition to these enzymatically catalysed lipidations, endogenous reactive metabolites formed by lipid peroxidation (called lipid-derived electrophiles (LDEs)), also bind covalently to cellular proteins.

**Table 1:** Major protein lipidations

Lipid	Modified residue (linkage)	Enzyme/chemical reaction	Mass spectrometry-based analysis
Octanoic acid (C8:0) 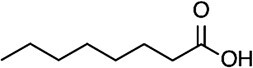	Serine (oxyester)	GOAT	*(* *67* *)*
Myristic acid (C14:0) 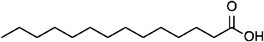	N-terminal glycine (amide) Lysine (amide)	NMT Unknown	*(* *2* *,* *3* *,* *20* *,* *52* *)*
Palmitic acid (C16:0) 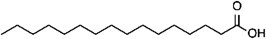	Cysteine (thioester), N-terminal cysteine (amide) Serine (oxyester)	ZDHHC HHAT LPCAT1	*(* *16* *,* *20* *,* *33* *,* *68* *,* *69* *)*
Palmitoleic acid (C16:1n7) 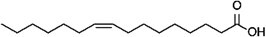	Serine (oxyester)	PORCN	*(* *70* *,* *71* *)*
Stearic acid (C18:0) 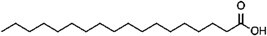	Cysteine (thioester)	DHHC	*(* *72* *,* *73* *)*
Oleic acid (C18:1n9) 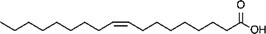	Lysine (amide)	Unknown	*(* *71* *)*
Lipoic acid 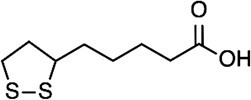	Lysine (amide)	LplA, LipA, LipB (*E. Coli*)	*(* *74* *,* *75* *)*
Farnesyl isoprenoid (C15) 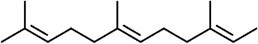 Geranylgeranyl isoprenoid (C20) 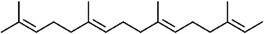	C-terminal cysteine (CAAX box motif) C-terminal cysteine (CAAX box motif)	FTase GGTase-I, II and III	*(* *20* *,* *36* *,* *76* *)*
Phosphatidylethanolamine (PE) 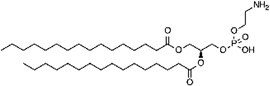 Phosphatidylserine (PS) 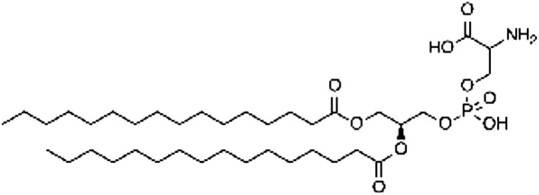	C-terminal glycine (amide) C-terminal glycine (amide)	ATG3 (for PE addition to LC3)	*(* *51* *,* *77* *,* *78* *)*
Cholesterol 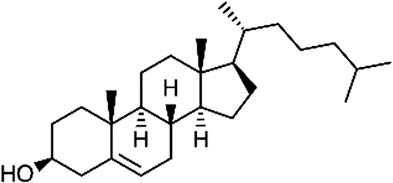	C-terminus (oxyester) aspartic acid (oxyester)	Autocatalytic mechanism (for SHH) Unkown for SMO	*(* *38* *,* *39* *)*
Glycosylphosphatidylinositol (GPI) 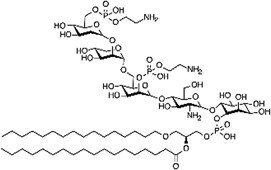	C-terminus (oxyester)	GPI transamidase (PIG-K, GPAA1, PIG-S, PIG-T, PIG-U)	*(* *79* *)*
Lipid-derived electrophile (LDE) 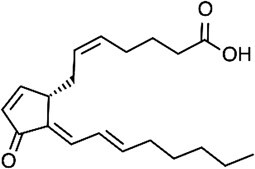 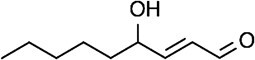	Nucleophilic residues (carbonyl, aldehyde)	Michael addition Schiff base reaction	*(* *40* *,* *43* *,* *44* *)*

Lipidation locally increases the hydrophobicity of proteins, altering their folding and their interactions with cellular membranes and other proteins. These changes in turn regulate subcellular localization, signaling networks, enzyme activity and intercellular communication *(**1**)*. Dysregulation of lipid modifications can result in diseases such as cancer, neurodegenerative diseases and infectious diseases *(**1**)*. Therefore, knowledge of proteins undergoing lipidation, as well as their lipid modification sites, is expected to provide insights into many aspects of lipid-related biology and potentially provide a basis for new therapeutic strategies that target protein lipidation *(**1**,**8**)*. For example, lonafarnib, an inhibitor of farnesyltransferase (FTase), is currently in clinical trial for Hutchinson–Gilford progeria syndrome, which is caused by a mutation in *LMNA* that produces the farnesylated aberrant lamin A protein *(**9**)*.

Bottom-up proteomics using liquid chromatography/tandem mass spectrometry (LC/MS/MS) is one of the most powerful strategies for global analysis of post-translational modifications (PTMs), including phosphorylation and acetylation. In bottom-up proteomics, proteins are extracted from tissues or cells and proteolytically digested into peptides. Then, the digested peptides are separated by LC and sequentially ionized by electrospray (electrospray ionization, ESI) and analysed by MS to determine their amino acid sequences *(**10**)*. Finally, peptides/proteins are identified by matching the peptide fragment ion MS/MS spectra to theoretical spectra generated from protein databases.

For large-scale analysis of PTMs, modified peptides need to be biochemically enriched from protein digests, since their abundance is relatively low compared to that of the unmodified counterparts. Typically, modified peptides are enriched prior to LC/MS/MS by using metal oxide (e.g. TiO_2_) affinity chromatography for phosphopeptides *(**11**)* or immunoprecipitation using anti-acetylated lysine antibody for lysine-acetylated peptides *(**12**)*. Such peptide-level enrichment is a critical step in pinpointing modification sites with regulatory potency. According to the UniProt database (https://www.uniprot.org/), protein lipidation is underrepresented compared to other major PTMs, including phosphorylation and acetylation (Figure 1); only 494, 201, 163 and 61 sites have been annotated for *N*-myristoylation at the protein N-terminus, *S*-palmitoylation at cysteine, *S*-geranylgeranylation at cysteine and *S*-farnesylation at cysteine, respectively. This is likely to be due to both biological and technical factors. Firstly, protein lipidation may be less frequent in biological systems compared to phosphorylation and acetylation. Secondly, from the technical aspect, the diversity of lipid structures and their physicochemical properties (e.g. increased hydrophobicity) makes it difficult to specifically enrich lipidated proteins/peptides. In addition, some types of lipidation, including cysteine *S*-palmitoylation, are labile in MS analysis *(**13**)*.

To investigate lipidated proteins, the metabolic incorporation of radiolabeled lipids has traditionally been utilized *(**14**)*. While this approach has identified various protein lipidations, a long autoradiographic exposure time is typically required, and the use of radioisotopes is potentially hazardous. Also, comprehensive identification of lipidated proteins is difficult since protein/peptide enrichment based on radiolabeling is not feasible. In recent years, MS-based proteomics has become a promising tool for global analysis of protein lipidation. For reversible lipidations, such as cysteine *S*-palmitoylation, the acyl-biotin exchange (ABE) method *(**15**,**16**)* has often been employed, in which the acyl group is chemically replaced with biotin and the resulting proteins are collected using avidin-immobilized beads. The bound proteins are then comprehensively identified by LC/MS/MS. Recently, large-scale analysis of various lipidations has been achieved by using lipid alkyne probes that are taken up by cells, followed by click chemistry-based affinity purification and LC/MS/MS analysis of proteins metabolically labeled with the lipid probes *(**17**)*. Liquid–liquid extraction (LLE) of endogenous lipidated peptides in combination with LC/MS/MS is also promising approach to capture a variety of protein lipidations in vivo *(**18*–*20**)*.

Here, we review proteomic methods for analysing lipidation, focusing on the two major approaches: 1) using chemical probes such as lipid alkyne probes and 2) using enrichment techniques for endogenous lipid-modified peptides. Both approaches are performed in the typical workflow of bottom-up proteomics. The differences between these two approaches are whether chemical probes are required and whether modified peptides are directly identified. The biological and functional significance and biochemistry of lipid modifications have been well reviewed elsewhere *(**1**,**22*–*25**)*.

## Global Analysis of Protein Lipidation Using Chemical Probes

In recent years, chemical proteomics has emerged as a powerful technology for the comprehensive analysis of protein lipidation. In particular, metabolic labeling of proteins in cultured cells with alkyne or azide-tagged lipid analogs is one of the most widely applicable methods to globally label and isolate lipidated proteins (Figure 2A). The size of the chemical tag is small, so that it minimally interferes with the substrate recognition and catalytic efficiency of lipid transferases, permitting the transfer of the analogs onto the target proteins. Once cellular proteomes are labeled with alkynylated (or azidized) lipids, reporter molecules and/or affinity handles are coupled to the probe-labeled proteins by copper-catalysed azide-alkyne cycloaddition (CuAAC, also known as click chemistry) or other bioorthogonal reactions *(**26**)*. Fluorescent molecules are typically used as a reporter to detect protein lipidation on SDS-PAGE gels, while biotin can be used to enrich and purify lipidated proteins in combination with streptavidin beads. The enriched proteins can be analysed by LC/MS/MS-based proteomics for global identification and quantification of lipidated proteins.

**Fig. 1:**
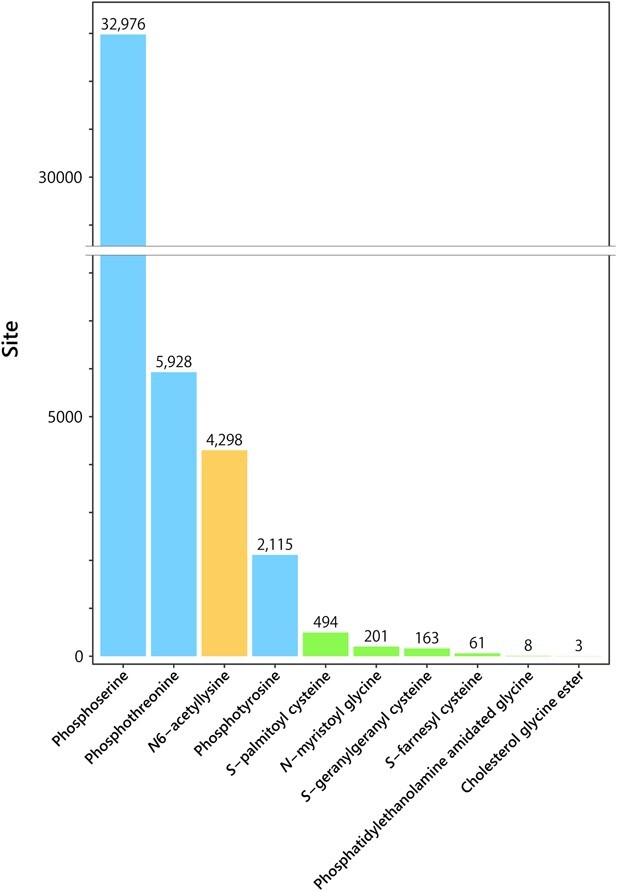
**Numbers of protein modification sites in humans reported in UniProt.** The numbers of modification sites in humans reported in the UniProt database (as of June 2023) are shown for selected modifications.

**Fig. 2:**
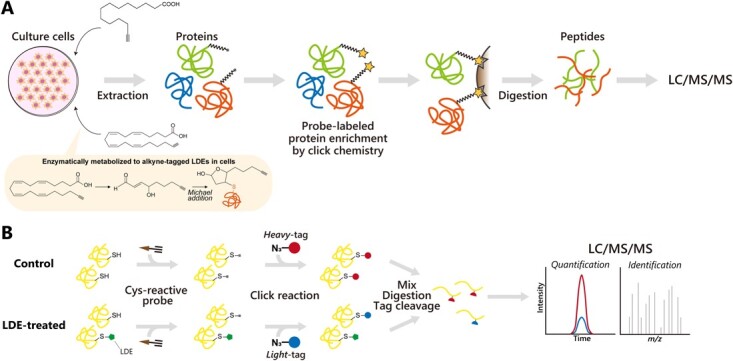
**Overview of proteomic identification of lipidated proteins using lipid alkyne probe.** (A) Alkyne-tagged lipid analogs are incorporated into the cell, and cellular proteins are modified by alkyne probes directly (top) or by alkyne-tagged LDEs that are generated from omega-alkynyl PUFAs by fatty acid oxygenases in the cell (bottom). Then, probe-labeled proteins are enriched using click chemistry, proteolytically digested and identified by LC/MS/MS. (B) Proteome-wide identification of targets of LDEs by Cys-reactive probe-based quantitative proteomics. After treating cells with LDE, cell lysates are labeled with a Cys-reactive probe, followed by the introduction of isotopically heavy or light biotin tags using click chemistry. After mixing control or LDE-treated proteomes in a 1:1 ratio, probe-modified proteins are captured by streptavidin beads, and sequential on-bead protease digestions are performed to yield probe-labeled peptides for LC/MS/MS analysis. Probe-modified peptides are identified based on the MS/MS spectrum, and light-to-heavy ratios of probe-modified peptides are quantified based on the MS1 peak intensity.

### Exploration of protein lipidation using alkyne or azide-tagged lipids

In a pioneering study, Hang et al. established a proof-of-concept of the use of omega-azido fatty acids for the global detection of protein fatty acylation in RAW264.7 macrophages *(**27**,**28**)*. By means of MS-based analysis, Kostiuk et al. identified 19 novel palmitoylated proteins in addition to 2 previously characterized ones in rat liver mitochondrial matrix *(**29**)*. A similar approach identified post-translational myristoylation of caspase-cleaved proteins during apoptosis *(**30**)*. Similar to azide analogs, omega-alkynyl fatty acids have also been used to identify fatty acylated proteins. 17-Octadecynoic acid (17-ODYA), which has been used as a P450 fatty acid omega-hydroxylase inhibitor, was employed as a clickable palmitic acid analog, and this enabled ~ 125 predicted palmitoylated proteins to be identified in Jurkat T cells *(**31**)*. Moreover, 17-ODYA metabolic pulse-chase labeling in combination with stable-isotope labeling by amino acids in cell culture (SILAC) *(**32**)* proteomics made it possible to profile the dynamics of palmitoylated proteins in mouse T-cell hybridoma cells *(**33**)*. Pulse-chase experiments using an omega-alkynyl fatty acid with 16 carbon atoms followed by fluorescence cell imaging of probe-labeled proteins revealed that the acylated proteins underwent rapid turnover with loss of the fluorescence signal within 3–6 h *(**34**)*, which is consistent with the findings in previous studies using radiolabeled fatty acids *(**35**)*.

Clickable lipid analogs have widely been applied to other lipidations, besides myristoylation and palmitoylation. Storck et al. developed alkynylated analogs of isoprenoid for global profiling of prenylated proteins in cells *(**36**)*. For protein cholesterylation, azide or alkyne-tagged cholesterol probes have been developed, and Sonic Hedgehog, a known target of cholesterylation, was detected by these probes *(**37**,**38**)*. Xiao et al. synthesized a different azide-tagged cholesterol analog to explore cholesterylated proproteins in cells, and mass spectrometric analysis revealed cholesterol modification of Smoothened (SMO), which is essential for Hedgehog signaling *(**39**)*.

### Chemical proteomic approaches to explore proteins modified by lipid-derived electrophiles

Polyunsaturated fatty acids (PUFAs), which are released from membrane phospholipids by phospholipase A_2_, are metabolized by fatty acid oxygenases such as lipoxygenases (LOXs) and cyclooxygenases (COXs) to form various kinds of lipid metabolites. Among them, fatty acid peroxides are further metabolized to lipid-derived electrophiles (LDEs) that covalently bind to cellular proteins. As with other lipidations described above, chemical proteomics approaches using azido or alkynyl derivatives of LDE have been applied for the global identification of protein targets of LDEs *(**40**)*. On the other hand, omega-alkynyl PUFAs can be metabolized by fatty acid oxygenases and thus can be used to trace the enzymatic oxidation and further metabolism of PUFAs *(**41**,**42**)*. Using omega-alkynyl PUFAs, we performed chemical proteomics to globally identify proteins modified by LDEs that are generated by fatty acid oxygenase in cells. We applied this method to mouse peritoneal macrophages and identified a series of protein targets of LDEs through a 12/15-LOX–catalysed reaction *(**43**)*. Pathway analysis revealed a significant enrichment of proteins involved in energy metabolism, and genetic deletion of 12/15-LOX resulted in altered glycolytic flux and mitochondrial respiration in mouse peritoneal macrophages *(**43**)*. Besides the enzymatic process, LDEs can also be generated by reactive oxygen species (ROS) that are produced under conditions of oxidative stress. Metabolic labeling of omega-alkynyl PUFAs in combination with SILAC proteomics identified protein targets of LDEs generated by ROS in RAW264.7 macrophages exposed to inflammatory stimuli *(**44**)*.

In addition to alkynylated lipid probes, an alternative chemical proteomics method using a cysteine (Cys)-reactive probe is also available to identify target proteins of LDEs. Among the 20 amino acids that compose proteins, cysteine has a uniquely high nucleophilicity, which makes it susceptible to modification by electrophilic compounds including LDEs. Thus, Cys-reactive iodoacetamide-based chemical probes can be used to map functional cysteines in complex proteomes (Figure 2B). Covalently acting small molecules including LDEs compete with the binding of Cys-reactive probes. Thus, enrichment of Cys-reactive probe-modified peptides in combination with quantitative proteomics enables global identification of target proteins of these electrophilic molecules as well as their modification sites by quantifying the inhibition of probe-modified peptides in response to the electrophilic molecules *(**45**,**46**)*. Using this approach, Wang et al. identified a series of cellular proteins that are targeted by exogenously added LDEs including 4-hydroxy-2-nonenal (HNE) and 15-deoxy-∆12,14-prostaglandin J_2_ (15d-PGJ_2_) *(**47**)*.

### Limitations of the chemical probe-based approaches

Chemical probes are limited to the major classes of lipidation mentioned above and may not capture all endogenous lipid modifications. Also, click chemistry-based affinity purification of alkyne or azide-tagged lipid-labeled proteins often provides only protein-level information (not peptide/site-level). In those approaches, biotin is appended with CuAAC, and on-beads peptide digestion is usually performed. In that process, the peptides containing modification sites remain bound to avidin beads even after digestion and thus cannot be detected by LC/MS/MS analysis. On the other hand, cleavable biotin tags have recently been developed, which allow selective release of the probe-modified peptides from the avidin beads to identify sites of modification *(**39**)*. Metabolic labeling with a chemical probe is mainly applied to cells and is not readily applicable *in vivo*, such as in animal tissues. This is possibly due to low tissue translocation and/or in vivo metabolism of chemical probes, which may result in an insufficient amount of lipid probes in the tissue to capture the modified protein.

Furthermore, chemical probes might not capture intrinsic lipidations due to structural differences from endogenous lipids. In addition, chemical probe-based metabolic labeling can cause off-target effects; for instance, metabolic labeling with a myristic acid analog is not entirely specific for myristoylation, as it also involves other targets, such as GPI-anchored proteins and palmitoylated proteins. Hence, additional evidence that affinity capture of myristoylated proteins is indeed inhibited by a specific *N*-myristoyltransferase (NMT) inhibitor is typically required to confirm bona fide targets. Similarly, the use of prenyltransferase inhibitors in combination with isoprenoid alkyne analogues has enabled the validation of targets of prenylation as well as evaluation of the potency of the inhibitors towards each target *(**36**)*.

## Global Analysis of Endogenous Lipidated Peptides/Sites

### Biochemical enrichment of endogenous lipidated peptides

**Fig. 3:**
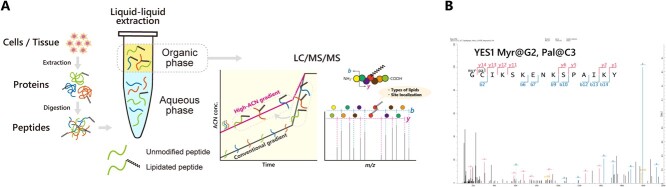
**Overview of identification of lipidated peptides using LLE in combination with a specialized LC gradient.** (A) Proteins are extracted from cells or tissues and proteolytically digested. Lipidated peptides are extracted by LLE and analysed by LC/MS/MS employing a specialized gradient focused on a higher ACN concentration range. The type of lipid and the modified site are identified based on the MS/MS spectrum. (B) MS/MS spectrum of YES1 protein N-terminal peptide with dual lipidation. The N-terminus is myristoylated, and the cysteine residue is palmitoylated.

Liquid–liquid extraction (LLE) has been commonly used to separate lipids and nonlipid species (e.g. salts, proteins, sugars) based on partitioning of lipids into an organic phase *(**48**,**49**)*. Similarly, LLE can be used to separate and enrich lipidated peptides from a protein digest. For example, ethyl acetate *(**18**,**19**)* and chloroform/methanol extraction followed by *n*-dodecyl-β-D-maltoside solubilization *(**50**)* were used for the isolation of hydrophobic lipidated peptides. Triton X-114-based phase partitioning was also shown to be effective for the separation of ubiquitinated phosphatidylethanolamine (PE) from unmodified ubiquitin *(**51**)*. Recently, Tsumagari et al. systematically evaluated organic solvents for LLE of lipidated peptides and found heptanol be especially suitable for the enrichment of myristoylated peptides *(**20**)* (Figure 3A). Tsumagari et al. demonstrated that LLE in combination with multiple digestion in parallel and a specialized LC gradient (described later) enabled identification of 75 myristoylation sites in HeLa cells (Figure 3A) *(**20**)*, which was greater than the number of myristoylated proteins previously identified using chemical probes *(**52**,**53**)*. Furthermore, this method allowed simultaneous enrichment of peptides with at least four forms of lipidation, *N*-myristoylation, *S*-palmitoylation, *S*-farnesylation, *S*-geranylgeranylation, as well as dually modified peptides with *N*-myristoylation and *S*-palmitoylation (Figure 3B) from HeLa protein digests. Collectively, LLE appears to have the potential for global analysis of a variety of lipidated peptides, including as-yet-unknown lipid modifications. Further, it can be applied to *in vivo* multicellular organisms including plants *(**2**,**3**)* and mammals *(**3**,**20**)*, which is one of the advantages of this method over chemical probe-based approaches *(**17**)*.

In addition to LLE, lipidated peptides can also be enriched using reversed-phase chromatography. Gustafsson et al. demonstrated that a 35-residue palmitoyl peptide was separated by using 60–75% methanol/ethanol as the initial solvent followed by elution with isopropanol, while most unmodified peptides were not retained on the C18 column under this condition *(**54**)*. Moreover, recently Ji et al. reported cysteine *S*-acylated peptides, including *S*-palmitoylated peptides, can be efficiently enriched using nanographite fluoride (nGF-SPE) *(**55**)*. If it is possible to simultaneously perform lipidated peptide enrichment and desalting, which is an essential process in sample preparation for bottom-up proteomics, this would be a simple and particularly useful technique.

### Choice and combination of proteases

In conventional bottom-up proteomics, trypsin (in combination with LysC) is a first choice for proteolytic digestion because it yields peptides with a suitable length for MS detection and peptides with C-terminal positive charges that enhance the solubility, ionization efficiency and/or MS/MS fragmentation efficiency of the ions. On the other hand, trypsin alone can be a limiting factor in peptide-centric proteomics, and thus multiple proteases, such as AspN, CluC, LysN and chymotrypsin, have been explored to expand the peptide sequence coverage within proteins *(**56**)*. In particular, chymotrypsin cleaves peptide bonds at the C-termini of hydrophobic amino acids (Tyr, Phe, Trp and Leu) and therefore is effective for the identification of membrane proteins *(**57**)*. Indeed, Tsumgari et al. demonstrated that the combination of three digestion patterns (trypsin/LysC, GluC, chymotrypsin) boosted the identification of *N*-myristoylated peptides approximately 1.4-fold in comparison to trypsin/LysC alone *(**20**)*. For example, chymotrypsin digestion enabled the identification of a dually modified peptide (YES1: G(*N*-Myr)C(*S*-Pal)IKSKENKSPAIKY) (Figure 3B) that contains 4 lysine residues (cleavage sites for trypsin/LysC) and 1 glutamic acid residue (a cleavage site for GluC) within the peptide, highlighting the value of employing multiple proteases in protein lipidation analysis.

### Other factors to be considered in sample preparation and LC/MS/MS

Among the various lipid modifications (Table 1), *S*-palmitoylation is a labile modification both in solution and in the gas phase. To minimize the loss of palmitoyl groups during sample preparation, palmitoylated peptides/proteins should be processed under neutral or slightly acidic conditions and at room temperature *(**13**)*. Instead of dithiothreitol (DTT), tris(2-carboxyethyl)phosphine (TCEP) is the preferred disulfide reducing agent, since it hardly affects palmitoyl protein analysis *(**13**,**20**)*. To enhance the detectability of highly hydrophobic lipidated peptides, a specialized gradient is needed in the LC/MS/MS measurement. While unmodified peptides or phosphorylated and acetylated peptides are typically eluted in the low acetonitrile (ACN) concentration range (i.e. 5–30%) of an LC gradient using a C18 column, extending the LC gradient to higher ACN concentration (e.g. ~ 80%) facilitates the separation and identification of lipidated peptides from complex peptide mixtures *(**2**,**3**,**20**)*. Although collision-induced dissociation (CID) is a commonly used peptide fragmentation technique, it appears to lead to facile loss of palmitoyl groups from *S*-palmitoylated peptides. Instead, electron transfer dissociation (ETD) might be an alternative fragmentation approach for palmitoyl peptides, as it produces extensive backbone fragmentation with minimum palmitoyl loss *(**13**)*. A systematic evaluation of fragmentation methods for lipidated peptides, including electron-transfer/higher-energy collision dissociation (EThcD) *(**58**)* and electron activated dissociation (EAD) *(**59**)*, is required to understand the modes of backbone fragmentation and neutral loss of lipid species that afford diagnostic ions for specific types of lipids.

### Limitations of the biochemical enrichment of lipidated peptides

One of the limitations of current biochemical lipidated peptide enrichment methods is the low efficiency in enrichment. While Tsumagari et al. identified protein N-terminal myristoylation sites using LLE on a large scale, only a few *S*-palmitoylated peptides were identified, even though a number of cysteine *S*-palmitoylation sites have been reported *(**20**)*. One potential reason for the difficulty in identifying palmitoylated peptides is their low stoichiometry. Given that protein lipidation mediates protein anchoring to cellular membranes, subcellular fractionation, for example to obtain plasma membrane and detergent-resistant membrane fractions, is also an effective way to enrich lipidated proteins prior to biochemical enrichment of lipidated peptides *(**2**,**3**)*.

## Perspective

Here, we have reviewed two major approaches for global analysis of protein lipidation based on 1) affinity purification of lipidated proteins labeled with lipid-mimicking chemical probes and 2) biochemical enrichment of endogenous lipidated peptides—both approaches have specific technical advantages and limitations and are complementary.

In living organisms, lipids exhibit tissue-, cell-type-, organelle-specific distributions *(**60**,**61**)*. Since protein lipidation is affected by the cellular metabolic state of lipids *(**62**,**63**)*, it is of interest to correlate the protein-lipidation states with spatial lipidomics and proteomics, since this may lead to the discovery of novel biological processes associated with local lipid environments. Notably, current advanced lipidomics technologies have established that lipids in living organisms are more complex and diverse than previously thought *(**61**,**64**)*. In this context, LLE-based methods that enrich various types of hydrophobic lipidated peptides should open up avenues for the discovery of new types of lipid modification by incorporating open modification search methods *(**65**,**66**)* that would allow us to explore unexpected modifications by means of mass spectrometry.
